# Monolayered Bi_2_WO_6_ nanosheets mimicking heterojunction interface with open surfaces for photocatalysis

**DOI:** 10.1038/ncomms9340

**Published:** 2015-09-11

**Authors:** Yangen Zhou, Yongfan Zhang, Mousheng Lin, Jinlin Long, Zizhong Zhang, Huaxiang Lin, Jeffrey C.-S. Wu, Xuxu Wang

**Affiliations:** 1 State Key Laboratory of Photocatalysis on Energy and Environment, Fuzhou University, Fuzhou 350002, China; 2 Department of Chemistry, Fuzhou University, Fuzhou 350108, China; 3 Fujian Provincial Key Laboratory of Theoretical and Computational Chemistry, Xiamen 361005, China; 4 Department of Chemical Engineering, National Taiwan University, Taipei 10617, Taiwan

## Abstract

Two-dimensional-layered heterojunctions have attracted extensive interest recently due to their exciting behaviours in electronic/optoelectronic devices as well as solar energy conversion systems. However, layered heterojunction materials, especially those made by stacking different monolayers together by strong chemical bonds rather than by weak van der Waal interactions, are still challenging to fabricate. Here the monolayer Bi_2_WO_6_ with a sandwich substructure of [BiO]^+^–[WO_4_]^2−^–[BiO]^+^ is reported. This material may be characterized as a layered heterojunction with different monolayer oxides held together by chemical bonds. Coordinatively unsaturated Bi atoms are present as active sites on the surface. On irradiation, holes are generated directly on the active surface layer and electrons in the middle layer, which leads to the outstanding performances of the monolayer material in solar energy conversion. Our work provides a general bottom-up route for designing and preparing novel monolayer materials with ultrafast charge separation and active surface.

## Abstract (web summary)

Although they tend to exhibit exciting optoelectronic behaviours, the difficulty in fabricating chemically bonded two-dimensional layered heterojunctions has hindered progress in the area. Here, the authors synthesize sandwich-substructured Bi_2_WO_6_ monolayers and investigate their photocatalytic activity.

## Graphical abstract

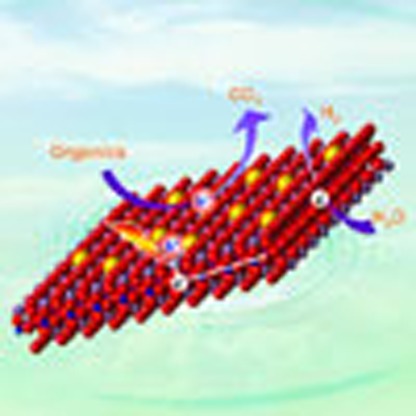

Semiconductor-based heterojunctions are able to facilitate fast charge separation, and thus have emerged as one of important strategies for creation of efficient solar energy conversion systems^1^,^2^,^3^,^4^. It is well known that a heterojunction in nature is the interface that occurs between two dissimilar crystalline semiconductors. The heterojunction effect on charge separation are thought to come from the built-in electric field forming at the atomically thin interface^1^. Compared with conventional powder heterojunction materials, the layered heterojunctions composed of different ultrathin two-dimensional (2D) materials have sparked widespread interests because of the well-defined heterostructures almost as thin as the interface of conventional heterojunctions. As great successes on monolayer materials have been achieved^5^,^6^,^7^, the stacks of different monolayers held together by van der Waals forces have been realized^8^,^9^,^10^,^11^,^12^,^13^,^14^,^15^. However, the layered heterojunctions composed of different monolayers stacked together by strong chemical bonds have been rarely studied. Since chemical bonding between monolayers can greatly enhance charge separation and even induce new energy band structures, the layered heterojunctions are considerably appealing^16^,^17^. Besides charge separation, exposure of active sites (mainly unsaturated atoms) is also key to affect the material performances in solar energy conversion^18^,^19^. The monolayers from van der Waals layered materials (with layers stacked by van der Waals forces) such as MoS_2_ have been found active sites only at the edge^20^,^21^,^22^. Post-processing methods such as defect engineering have often been applied for creating more active sites on these 2D materials^23^,^24^,^25^. Aurivillius phase oxides, as complex layered materials, consist of alternate stacking of [Bi_2_O_2_] layers and perovskite-type layers with oxygen atoms shared between layers. This means that such a monolayer material maybe regarded as the stacks of different monolayer oxides by chemical bonds, and may possess oxygen-depleted surfaces that expose a huge number of active sites.

It is well known that monolayer materials maybe achieved on a small scale by mechanical exfoliation or chemical vapour deposition^26^,^27^,^28^. However, the practical and large-scale synthesis of freestanding monolayers in liquid phase is irreplaceable for many applications. Up to now, most monolayer materials are produced by liquid exfoliation of the van der Waals layered materials, such as graphene, boron nitride (BN) and transition metal dichalcogenides^29^,^30^,^31^. However, it is difficult to exfoliate the layered materials with layers stacked by strong chemical bonds. The alternative method, a bottom-up approach, has been used to produce multilayer nanosheets, but rarely succeed in monolayer materials^32^,^33^. The key to preparation of such monolayers by the bottom-up route is to keep monolayers from stacking together.

We develop a cetyltrimethylammonium bromide (CTAB)-assisted bottom-up route to fabricate such freestanding monolayer materials. The successfully synthesized monolayer Aurivillius oxide Bi_2_WO_6_ has a sandwich substructure of [BiO]^+^–[WO_4_]^2−^–[BiO]^+^, mimicking heterojunction interface with space charge. The Br^−^ ions from CTAB strongly adsorb on the monolayers surfaces and thus make the monolayers negatively charged. In the self-assembly process, stacking of the monolayers is blocked by Coulomb repulsion forces and the hydrophobic chains of CTA^+^ ions. The Br^−^ ions on surface induce a decrease in the bandgap energy of the monolayers. The surface Bi atoms are coordinatively unsaturated and thus the monolayers surfaces expose a huge number of active sites. On irradiation, holes are directly generated on the active surfaces and electrons in the middle layer, leading to ultrafast charge separation. Both the highly active surface and the ultrafast charge separation contribute to the superior photocatalytic performances of the monolayer material. In the presence of positive dyes, the visible light activity of the monolayers can be greatly enhanced due to photosensitization of the strongly adsorbed dye.

## Results

### Self-assembly of Bi_2_WO_6_ monolayers

**Figure 1:**
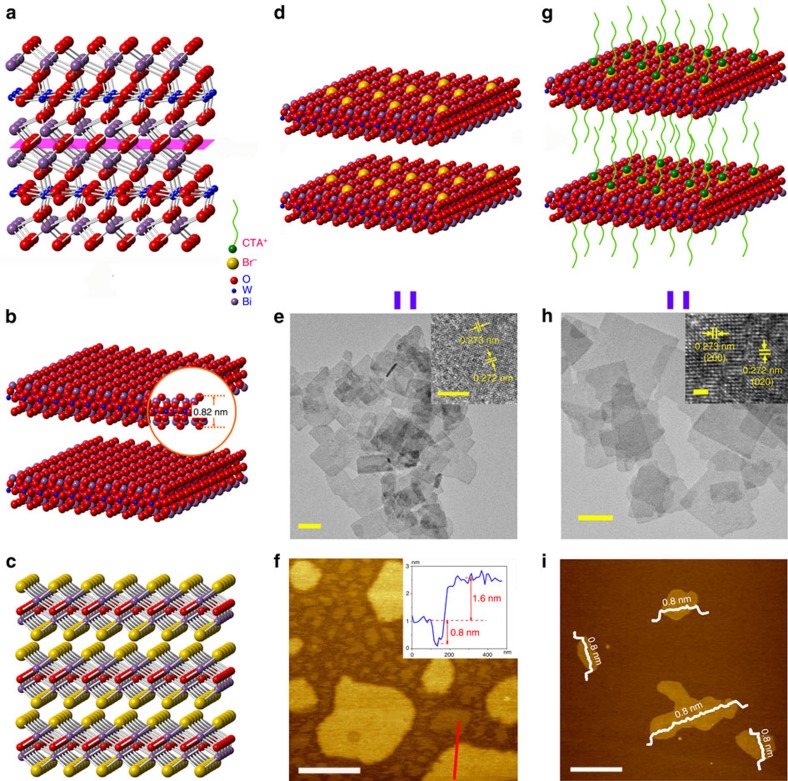
Synthesis of monolayer Bi_2_WO_6_. (**a**) Crystal structure of Bi_2_WO_6_. (**b**) Structure of pristine monolayer Bi_2_WO_6_. (**c**) Crystal structure of BiOBr. (**d**) Formation mechanism of the monolayer Bi_2_WO_6_ with Br^−^ ions assistance. (**e**) TEM image of the synthesized Bi_2_WO_6_ with Br^−^ ions assistance; inset is the corresponding HRTEM image. (**f**) AFM image of the monolayer Bi_2_WO_6_ prepared by Br^−^ ions assistance; inset is the corresponding height profile on the red line. (**g**) Formation mechanism of the monolayer Bi_2_WO_6_ with CTAB assistance. (**h**) TEM image and the corresponding HRTEM image of the Bi_2_WO_6_ prepared with CTAB assistance. (**i**) AFM image of the monolayer Bi_2_WO_6_ prepared by CTAB assistance. Scale bar, 500 nm (**f**,**i**), 50 nm (**e**,**h**), 5 nm (insets in **e**) and 1 nm (insets in **h**).

As shown in Fig. 1a, Bi_2_WO_6_ is a layered material built up of [Bi_2_O_2_] layers and corner-shared WO_6_ octahedral layers. Monolayer Bi_2_WO_6_ has two possible configurations: [BiO]^+^–[WO_4_]^2−^–[BiO]^+^ (sandwich substructure) and [Bi_2_O_2_]^2+^–[WO_4_]^2−^ (non-sandwich substructure). Density functional theory (DFT) calculations show that the surface energy of the sandwich configuration is 0.86 J m^−2^, notably smaller than that of the non-sandwich substructure (2.27 J m^−2^). It can be deduced accordingly that the monolayer Bi_2_WO_6_ has the sandwich structure, as presented in Fig. 1b.

As mentioned above, the monolayer Bi_2_WO_6_ exposes coordinatively unsaturated Bi atoms on surface (Fig. 1b). This induces strong interaction between the monolayers and therefore makes the monolayers to stack easily into multilayers. So far no monolayer nanosheets have been achieved through bottom-up route^33^,^34^. Obviously, weakening the interaction between the monolayers is a key to successfully obtain the monolayer material. It is well known that BiOBr is a typical layered material consisting of (Br–Bi–O–Bi–Br) slices stacked together by van der Waal interaction, in which the Br atoms terminate the dangling bonds of slices (Fig. 1c)^35^. When Br^−^ ions were introduced in the bottom-up synthesis system for Bi_2_WO_6_, they would bond to the monolayers surfaces to terminate the dangling bonds partly and make the surface negatively charged, which would suppress the stacking of the monolayers, as shown in Fig. 1d.

As expected, the monolayer Bi_2_WO_6_ was achieved when KBr as a Br^−^ source was added to the hydrothermal reaction system of Bi(NO_3_)_3_·5H_2_O and Na_2_WO_4_·2H_2_O (Br/Bi atomic ratio is 1:20). The X-ray diffraction patterns of the resulted sample are shown in Supplementary Fig. 1. All the diffraction peaks can be indexed to an orthorhombic Bi_2_WO_6_ phase with lattice parameters *a*=5.457 Å, *b*=5.436 Å and *c*=16.427 Å (JCPDS No. 73-2020). The transmission electron microscopy (TEM) image in Fig. 1e exhibits the synthesized Bi_2_WO_6_ with a sheet-shaped structure. Inset in Fig. 1e gives the corresponding high-resolution transmission electron microscopy (HRTEM) image. The marked interplanar spacings of (200) and (020) planes indicate that the Bi_2_WO_6_ nanosheets expose {001} facets. The atomic force microscopic (AFM) image and the corresponding height histograms of the nanosheets are presented in Fig. 1f and Supplementary Fig. 2, respectively. It can be seen that the thinnest nanosheets have a thickness of ca. 0.8 nm, which agrees well with that of monolayer Bi_2_WO_6_ sheet along [001] direction (corresponding to 1/2 of the unit cell size in *c*-axis direction). This shows that the Bi_2_WO_6_ monolayers can be achieved by a Br ions-assisted hydrothermal method. Unfortunately, some bilayer nanosheets are observed in the products.

CTAB is a long-chain cationic surfactant containing Br^−^ ions. When CTAB instead of KBr was used in the synthesis, the CTA^+^ ions would adsorb on the surface with bonding of Br^−^ ions to the monolayers surfaces. Thus, the extra surface repulsion from the hydrophobic chains of CTA^+^ further prevents stacking of the monolayers, as showed in Fig. 1g. In this case, the high-quality monolayer Bi_2_WO_6_ was successfully prepared. The X-ray diffraction result reveals that the obtained sample is pure phase Bi_2_WO_6_ (Supplementary Fig. 1). The sample shows weaker X-ray diffraction peaks than that prepared with only Br^−^ ions, indicating the smaller size of the sample. The TEM image (Fig. 1h) displays sheet-shaped structures of the sample. It can be seen from the HRTEM image (inset of Fig. 1h) that marked interplanar spacings of (200) and (020) planes appear on the sample, indicating the {001} exposed facets of the nanosheets. The AFM image in Fig. 1i shows that the thickness of nanosheets is very close to that of monolayer Bi_2_WO_6_ slab (the height histograms in Supplementary Fig. 3). These results illustrate that the well-defined Bi_2_WO_6_ monolayers were successfully prepared by the one-pot CTAB-assisted hydrothermal process.

In addition, a control synthesis with neither KBr nor CTAB assistance was also performed for comparison. Only the Bi_2_WO_6_ nanocrystals were obtained in this case. Both the TEM image (Supplementary Fig. 4) and the HRTEM image (Supplementary Fig. 5) show that the sample is 20 nm diameter near-spherical aggregation consisting of 8 nm diameter nanocrystals. The X-ray diffraction pattern of nanocrystals is the same as that of the monolayers obtained by CTAB assistance (Supplementary Fig. 6), indicating the same crystal phase. Above results show that the Br^−^ and CTA^+^ are responsible for the formation of monolayer Bi_2_WO_6_, which were further discussed in Supplementary Figs 7–9.

### Chemical compositions of Bi_2_WO_6_ monolayers

**Figure 2:**
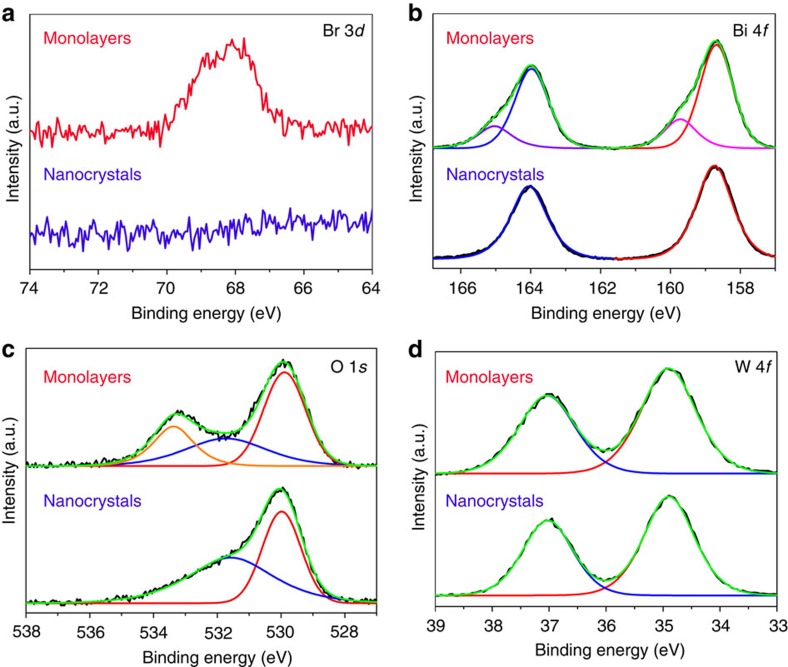
XPS of Bi_2_WO_6_ samples. (**a**) Br 3*d*, (**b**) Bi 4*f*, (**c**) O 1*s* and (**d**) W 4*f*.

X-ray photoelectron spectroscopic (XPS) characterizations were conducted to reveal the chemical compositions of the monolayer Bi_2_WO_6_ (ref. 36). The Br 3*d*, Bi 4*f*, W 4*f* and O 1*s* XPS spectra of the Bi_2_WO_6_ monolayers and nanocrystals are shown in the Fig. 2. For the monolayers, a strong Br 3*d* peak is observed at the binding energy of 68.5 eV, which suggests that the Br^−^ ions from CTAB are bonded to the surface Bi atoms of the monolayer^37^. Compared with the nanocrystals, the monolayers present two asymmetric Bi 4*f* peaks (Fig. 2b), indicating the different chemical states of Bi for the two samples. Both the asymmetric Bi 4*f* peaks can be deconvolved into two peaks. The shoulder peaks (165.0 eV for Bi 4*f*_5/2_, and 159.9 eV for and Bi 4*f*_7/2_) occur at higher binding energies, indicating higher electropositive Bi appearing in the monolayers. This can be indicative of some Bi atoms bonded with the surface Br atoms^37^. Figure 2c is O 1*s* spectra, two peaks at 529.9 eV and 531.7 eV are observed for both samples, which are assigned respectively to lattice oxygen and bridging hydroxyls^38^. But the peak at 533.3 eV belonging to physisorbed water only appears for the monolayers^39^. This is because the open metal sites on the monolayers surfaces have strong interaction with surrounding water. Figure 2d shows that the W 4*f* spectra are almost comparable for two samples, which can be explained by [WO_4_]^2−^ locating at the middle layers of the monolayers and hardly affected by the surface Br atoms.

Quantitative XPS analysis shows that the ratios Br/Bi/W are 0.2/2.1/1.0 in the monolayer Bi_2_WO_6_ sample, of which the Bi/W ratio is almost identical to that of the nanocrystals. This result implies that the Br^−^ ions are non-stoichiometrically coordinated to [BiO]^+^ layers together with the CTA^+^. An additional experiment shows that the CTA^+^ ions on the monolayers surfaces can be removed by washing (Supplementary Fig. 10). H^+^ ions maybe substitute the CTA^+^ ions for keeping charge balance of the monolayer in the washing process. The residual amount of CTA^+^ ions on the surface are very low and no obvious influence was observed on the photocatalytic activity of the monolayers (Supplementary Figs 11–12).

### Synthesis of other monolayer materials

To verify the universal applicability of this self-assembly route, preparation of monolayer Bi_2_O_2_CO_3_ was studied also in the same conditions. The result shows that the monolayer Bi_2_O_2_CO_3_ with a thickness of 0.55 nm was successfully obtained in the presence of CTAB, as showed in the Supplementary Figs 13–15. Besides Bi_2_WO_6_ and Bi_2_O_2_CO_3_, the Aurivillius phase layered oxides (general formula Bi_2_A_*n*−1_B_*n*_O_3*n*+3_; A=Ca, Sr, Ba, Pb, Bi, Na, K and B=Ti, Nb, Ta, Mo, W, Fe), an important class of functional materials including photocatalysts, superconductors, and ferroelectric materials^40^,^41^,^42^, have the common feature of containing [Bi_2_O_2_] layers in crystal structure. Thus, a huge number of monolayer Aurivillius materials can be formed by the similar mechanism of self-assembly.

### Band gap of the monolayer Bi_2_WO_6_

**Figure 3:**
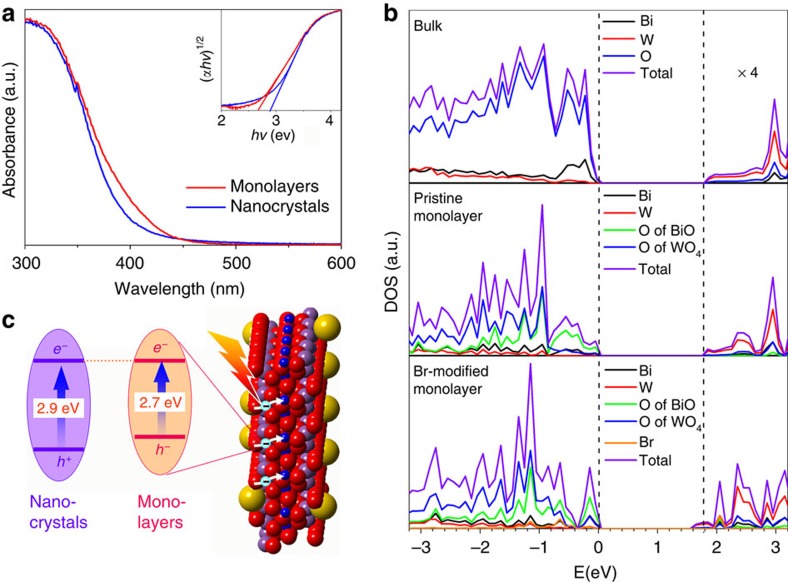
Bandgap states of Bi_2_WO_6_ samples. (**a**) Diffuse reflectance ultraviolet–visible spectra of Bi_2_WO_6_ samples. (**b**) Calculated density of states (DOSs) of Bi_2_WO_6_ bulk, pristine monolayer and Br-modified monolayer. The Fermi level is taken as the energy zero. (**c**) Band energy diagrams of Bi_2_WO_6_ nanocrystals and monolayers. W, O, Bi and Br atoms are represented as blue, red, purple and yarrow spheres, respectively.

Figure 3a shows ultraviolet–visible diffuse reflectance spectrum of the Bi_2_WO_6_ samples. The bandgap energies of the samples were estimated from the plots of (*αhv*)^1/2^ versus the energy of absorbed light (inset of Fig. 3a)^43^. The bandgap energy of the monolayers is ca. 2.7 eV, lower than that of the nanocrystals by 0.2 eV. To understand the differences, band structures of the bulk (multilayers), pure monolayer (unmodified) and Br-modified monolayer of Bi_2_WO_6_ were investigated theoretically (Supplementary Fig. 16). The corresponding densities of states are presented in Fig. 3b. Although the bandgaps from DFT calculations are usually smaller than that from the experimental determination, the DFT calculations often provide important insights into the physicochemical properties of the materials^43^,^44^. For the unmodified monolayers and the bulk, the top of valence band and the bottom of conduction band are mainly originated from hybrid Bi 6*s*–O 2*p* orbital and W 5*d* orbital, respectively (Fig. 3b). However for the Br-modified monolayers, the bottom of conduction band is still originated from W 5*d* orbital, but the top of valence band is composed of the hybridized orbital of Bi 6*s*, O 2*p* and Br 4*p* (Fig. 3b). Due to more negative potential of their top of valence, the Br-modified monolayers show a narrower bandgap than the unmodified monolayers and the nanocrystals.

The sandwich substructure of [BiO]^+^–[WO_4_]^2−^–[BiO]^+^ of monolayer Bi_2_WO_6_ simulates the heterojunction interface with space charge that promotes separation of the photogenerated carriers in the interface, as shown in Fig. 3c. It should be noted that the bottom of conduction band is originated from W 5*d* orbital located in the middle layer, and the top of valence band is mainly composed of O 2*p* orbital from the surface [BiO]^+^ layers (Fig. 3b). On irradiation, holes and electrons can be generated, respectively, on the surface layer and in the middle layer and thus are separated directly. In brief, for the monolayer Bi_2_WO_6_, the sandwich substructure and the Br surface modification lead to decrease of the bandgap and enhancement of the photogenerated charges separation, which are very favourable for photocatalysis.

### Open surface of the monolayer Bi_2_WO_6_

**Figure 4:**
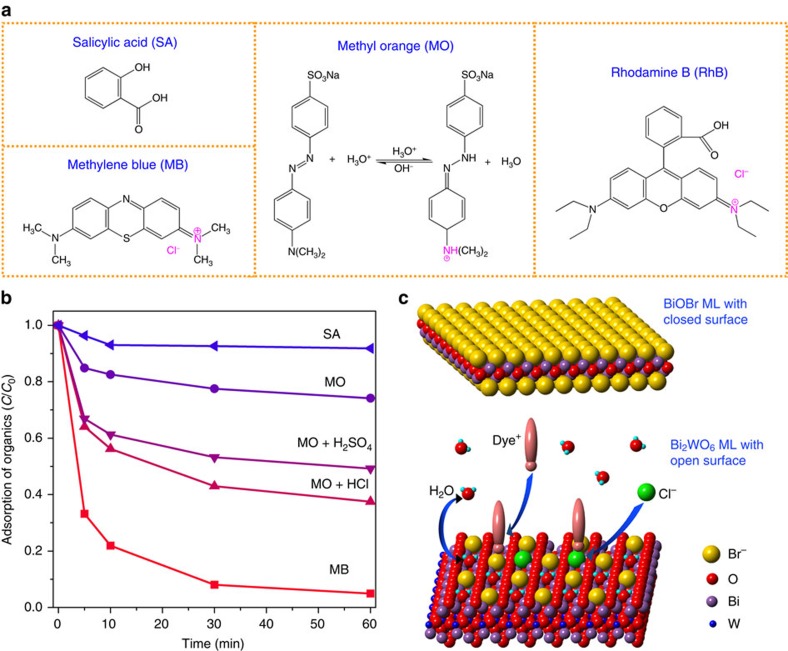
Open surface of the Bi_2_WO_6_ monolayers. (**a**) Molecular structures of various organic contaminants. (**b**) Adsorption of various organic pollutants over the Bi_2_WO_6_ monolayers. (**c**) Schematic diagram showing the interactions between the open surface of monolayer Bi_2_WO_6_ and surrounding species.

Different from most reported monolayer materials with closed and inert surfaces^21^, the as-synthesized monolayer Bi_2_WO_6_ possesses oxygen-depleted and highly active surface. Moreover, the Br^−^ ions on surfaces cause the monolayers negatively charged, as proved by the zeta potential in Supplementary Table 1. Therefore, the monolayers can selectively adsorb positively charged species. Figure 4b shows the adsorption performances of various organic pollutants over the Bi_2_WO_6_ monolayers. Salicylic acid, as a neutral species, is hardly adsorbed by the monolayers. Methyl orange is positively charged in acid medium (Fig. 4a), and therefore its adsorption is enhanced by changing solution pH from 7 to 3 using H_2_SO_4_ or HCl. Similar to Br^−^ ions, Cl^−^ could also adsorb at the open metal sites on surface and make the monolayers more negatively charged, as shown in Fig. 4c. Hence, the methyl orange adsorption can be promoted more effectively by HCl than by H_2_SO_4_. Methylene blue molecules containing both Cl^−^ ions and positive ammonium groups are adsorbed almost entirely. Owing to the open surfaces, the monolayers show much better ability for adsorption of Rhodamine B (RhB; 7.3 mg g^−1^) than the nanocrystals (0.19 mg g^−1^), though the specific surface area of the monolayers (43 m^2^ g^−1^) is less than twice that of the nanocrystals (28 m^2^ g^−1^). All above results confirm that the surface of monolayer Bi_2_WO_6_ is open and can strongly interact with surrounding species such as water, anions Cl^−^ or Br^−^ and positively charged organics (Fig. 4c).

### Photocatalytic performance of the monolayer Bi_2_WO_6_

**Figure 5:**
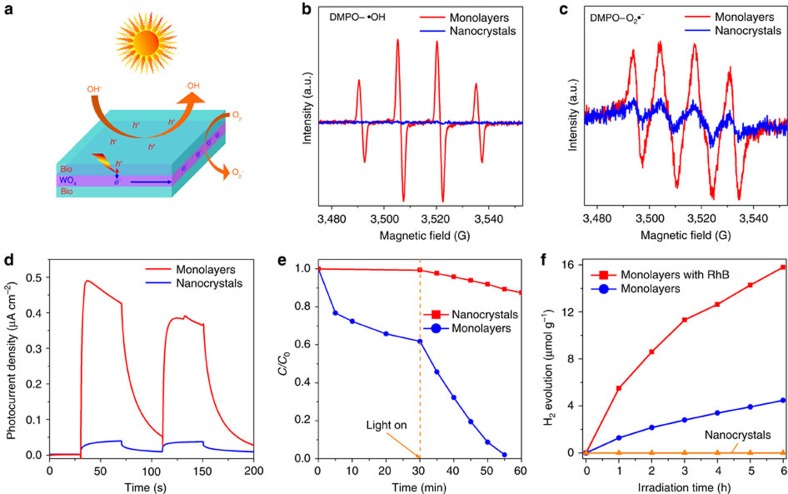
Photocatalytic performances of Bi_2_WO_6_ samples. (**a**) Schematic illustration of photocatalytic mechanism over the monolayer Bi_2_WO_6_. (**b**,**c**) Electron spin resonance signals of DMPO-·OH^−^ adducts and DMPO-O_2_·^−^ adducts produced by Bi_2_WO_6_ samples under visible light irradiation (*λ*≥420 nm). (**d**) Photocurrent of Bi_2_WO_6_ samples under visible light illumination. (**e**) Photodegradation of RhB over Bi_2_WO_6_ samples (*λ*≥420 nm). (**f**) Visible-light-driven photocatalytic H_2_ evolution over the nanocrystals, monolayers and RhB sensitized monolayers (*λ*≥420 nm); 0.3 wt% of Pt was loaded.

Since the holes are photogenerated directly on the open surface [BiO]^+^ layers (Fig. 5a), the monolayers surfaces are highly active for photocatalytic oxidation reactions, such as the oxidation of OH^−^ to ·OH. This feature was verified by the 5, 5-dimethyl-1-pyrroline-N-oxide (DMPO) spin-trapping electron paramagnetic resonance (EPR) technique (Fig. 5b). The ·OH radicals signal is very weak for the nanocrystals under visible light illumination, which is consistent with the previously observation for Bi_2_WO_6_ samples^45^. However, for the monolayer Bi_2_WO_6_, the ·OH radicals signal is much stronger, implying the superior activity of the monolayers for the photocatalytic oxidation reactions. On the other hand, the photoelectrons are formed in the middle layer where they transfer to the edge of monolayers for reduction reactions. Figure 5c shows that the reduction activity of the monolayers for O_2_ to O_2_^−^ is enhanced likewise, compared with that of the nanocrystals, although the enhancement is lower for the reduction than for the oxidation. Figure 5d is comparison of photocurrents between the monolayers and nanocrystals. It can be seen that the photocurrent from the monolayers is about 10-fold higher than that from the nanocrystals. This indicates that the monolayers mimic the heterojunction interface to result in the ultrafast charge transfer and separation. As for the little decrease of photocurrent on the monolayers with irradiation time, this can be because the photogenerated electrons formed in the middle layer need to undergo a long-distance transfer towards the edge, electron accumulation and partial recombination with holes may occur simultaneously with irradiation time.

The photocatalytic performances of Bi_2_WO_6_ samples for photodegradation of RhB were evaluated under visible light illumination (*λ*≥420 nm). As shown in Fig. 5e, the monolayers show much higher visible-light-driven photocatalytic activity than the nanocrystals. Ninety-eight percent of RhB are degraded using the monolayers as a photocatalyst within 25 min, while only 13% RhB are degraded using the nanocrystals within 30 min. Moreover, the monolayers can be efficiently recycled and reused without appreciable loss of activity (Supplementary Figs 17–18). Besides, the monolayers show also an excellent photocatalytic activity for H_2_ production from water solution under visible light. Figure 5f shows the H_2_ evolution over the nanocrystals, monolayers and RhB sensitized monolayers (*λ*≥420 nm) with 0.3 wt% Pt loading as a function of time. The H_2_ evolution activity of Bi_2_WO_6_ has been reported to be very low (1.6 μmol h^−1^ g^−1^) even under a 450 W high pressure mercury lamp^46^. And the Bi_2_WO_6_ has been considered to have no activity for visible-light-driven H_2_ production^47^. As shown in Fig. 5f, the nanocrystals show indeed no H_2_ evolution activity, but surprisingly the monolayers exhibit a good H_2_ evolution activity under visible light. As adding RhB into the reaction system, ca. fourfold higher activity is observed due to the sensitization effect of RhB. Both the ultrafast charge separation and the highly active surface make the monolayers possess superior performances in solar energy conversion than other nanostructures composed of two or more layers (Supplementary Figs 19–21).

## Discussion

In summary, the freestanding monolayer Bi_2_WO_6_ can be successfully synthesized via a CTAB-assisted self-assembly route. The as-synthesized monolayer Bi_2_WO_6_ has a sandwich substructure of [BiO]^+^–[WO_4_]^2−^–[BiO]^+^. The Bi atoms in the surface [BiO]^+^ layers are coordinatively unsaturated and thus the monolayers can adsorb Br^−^ ions to avoid stacking into multilayer. The monolayer Bi_2_WO_6_ shows oxygen-depleted surface, and has a narrower bandgap than the nanocrystals. The sandwich structure simulates the heterojunction interface with space charge to facilitate the ultrafast separation of photogenerated carriers. On irradiation, holes are generated directly on the highly active surfaces, resulting in the excellent activities for the photodegradation of RhB. More interestingly, the monolayering allows Bi_2_WO_6_ to have a visible-light-driven H_2_ evolution activity, which can be efficiently enhanced by dye sensitization. This work provides a simple and efficient bottom-up route to prepare novel monolayer materials with ultrafast charge separation and highly active surface.

## Methods

### Materials synthesis

In the preparation of Bi_2_WO_6_ monolayers, the start materials 1 mmol Na_2_WO_4_·2H_2_O, and 2 mmol Bi(NO_3_)_3_·5H_2_O and 0.05 g CTAB were added in 80 ml deionized water. After 30 min stirring, the mixed solution was poured into a 100 ml Teflon-lined autoclave. Then the autoclave was sealed into a stainless steel tank and treated at 120 °C for 24 h. Finally, the product was collected and washed several times with deionized water and dried at 60 °C in air for 10 h. In the preparation of monolayer Bi_2_WO_6_ companied with few layers, 0.1 mmol KBr instead of CTAB was added in the mixture, while keeping other parameters unchanged. The Bi_2_WO_6_ nanocrystals were prepared without CTAB and KBr assistances.

Bi_2_O_2_CO_3_ monolayers were prepared by the same route. A total of 2 mmol Bi(NO_3_)_3_ and 0.05 g CTAB were added in 80 ml of 1 M HNO_3_ solution. After the mixture became a clear solution, 10 mmol Na_2_CO_3_ was added and a white precipitate appeared immediately. This suspension was magnetically stirred for 30 min to complete the precipitation reaction. Then the suspension was poured into a 100 ml Teflon-lined autoclave and the autoclave was sealed into a stainless steel tank. The sample was treated at 60 °C for 24 h. Then the reactor was naturally cooled to room temperature. The obtained sample was collected and washed several times with deionized water and dried at 60 °C in air for 10 h.

### Materials characterization

The X-ray diffraction patterns were recorded on a Bruker D8 Advance X-ray diffractometer with Ni filtered Cu Kα radiation at 40 kV and 40 mA. Ultraviolet–visible diffuse reflectance (ultraviolet–visible diffuse reflectance spectrum) spectra were obtained with a self-supporting sample disk on a ultraviolet–visible spectrophotometer (Cary 500), where BaSO_4_ was used as a reflectance standard. TEM images were obtained using a JEOL model JEM 2010 EX instrument at an accelerating voltage of 200 kV. AFM images were recorded using Agilent 5,500 AFM (Agilent Technologies, USA). All the images were acquired using tapping mode under ambient conditions (ca. 40−50% relative humidity, 25 °C temperature). The used Si cantilevers/tips (Bruker) have a spring constant of 40 N m^−1^ and a resonance frequency of 300 kHz. During an AFM experiment, sample was dispersed in ethanol using an ultrasonic bath for 20 min and then the dispersion was diluted in ethanol. A drop of the above diluted dispersion was deposited on a new cleaved mica surface and dried in air. The instrument parameters (set point, amplitude, scan size, scan speed and feedback control) were adjusted for the best resolution of images. Electron spin resonance spectra were obtained over Bruker ESP 300 E electron paramagnetic resonance spectrometer.

### Activity evaluation

The photodegradation of dyes were performed in a glass vessel under visible-light irradiation by a 300 W Xe lamp with a 420 nm cutoff filter. Twenty milligram of the photocatalyst was added into 80 ml RhB (10 μmol l^−1^) solution. The dispersion was stirred in the dark for 30 min to reach the adsorption equilibrium. Then the visible light was turn on for the photodegradation tests. Three millilitres of the sample solution were taken at given time intervals and separated by centrifugation. The residual concentration of the organics in solution was analysed using a Varian Cary 50 Scan ultraviolet–visible spectrophotometer. The selective adsorption experiments were performed by the same method. The methyl orange (20 p.p.m.), methylene blue (10 p.p.m.) and salicylic acid (500 μmol l^−1^) solutions were used. 50 mg of the catalyst was used to test the adsorption performances.

The photocatalytic H_2_ production of samples under visible light irradiation was conducted in a Pyrex reactor connected with a gas-closed circulation system and a vacuum system. In a typical experiment, 20 mg catalyst was dispersed in 100 ml deionized water containing 0.4 g ethylenediamine tetraacetic acid (EDTA) as a sacrificial reagent. This system was evacuated for 30 min to remove air prior to irradiation. A 300-W Xe lamp with an optical cutoff filter (*λ*≥420 nm) was used as a light source. The produced hydrogen was quantified by gas chromatography (Shimadzu GC-8A, TCD, Ar carrier). For the RhB sensitization system, RhB (10 μmol l^−1^) was contained in solution.

### Calculation details

First-principles DFT calculations were carried out utilizing the Vienna *ab initio* simulation package^48^,^49^. The Perdew–Wang-type (PW91) exchange–correlation functional was employed to study the energies and structures of different Bi_2_WO_6_ systems. Vanderbilt ultrasoft pseudo-potentials were used to describe the interactions between the ion cores and valence electrons for all atoms, and the kinetic cutoff energy was set to 400 eV. In the calculations, the convergence energy threshold for self-consistent iteration was set to 10^−4^ eV per atom, along with the residual atomic forces were <0.03 eV Å^−1^. For the bulk Bi_2_WO_6_, the optimized lattice constants are *a*=5.480 Å, *b*=5.468 Å and *c*=16.760 Å, which agrees well with the experimental values of *a*=5.457 Å, *b*=5.436 Å and *c*=16.427 Å. A (2 × 2) periodic slab model was adopted to simulate different Bi_2_WO_6_ nanosheets, which were constructed from the Bi_2_WO_6_ (001) surface. During the structural optimization, the positions of all atoms and the lengths of two translation vectors of the nanosheet were allowed to relax. The spacing between the adjacent slabs was set about 10 Å, and a (3 × 3 × 1) Monkhorst–Pack *k*-point mesh was used for integration in the reciprocal space. To investigate the thermodynamic stability of different Bi_2_WO_6_ nanosheets, the surface free energy (*γ*) was calculated, which is defined as following,


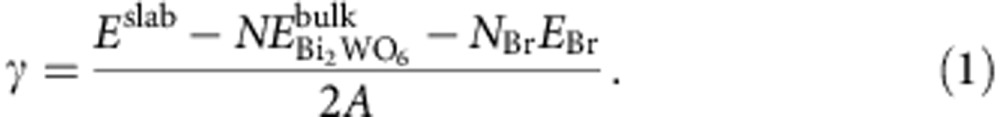


where *E*^slab^ is the total energy of the slab, 
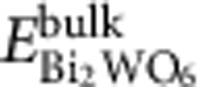
 is the energy of per Bi_2_WO_6_ unit in the bulk, *N* is the number of Bi_2_WO_6_ unit found in the slab, *N*_Br_ is the number of Br atom, *E*_Br_ is the ground state energy of Br atom, and *A* is the area of the slab surface.

## Additional information

**How to cite this article:** Zhou, Y. *et al*. Monolayered Bi_2_WO_6_ nanosheets mimicking heterojunction interface with open surfaces for photocatalysis. *Nat. Commun.* 6:8340 doi: 10.1038/ncomms9340 (2015).

## Supplementary Material

Supplementary Information Supplementary Figures 1-21, Supplementary Table 1 and Supplementary Reference
