# Supplementary material for: Monolayered Bi2WO6 nanosheets mimicking heterojunction interface with open surfaces for photocatalysis
Source: Nat Commun. 2015 Sep 11;6:8340. doi: 10.1038/ncomms9340 (PMC4647850; doi:10.1038/ncomms9340)
Supplement: Supplementary Information — Supplementary Figures 1-21, Supplementary Table 1 and Supplementary Reference [file ncomms9340-s1.pdf]

Supplementary Figures

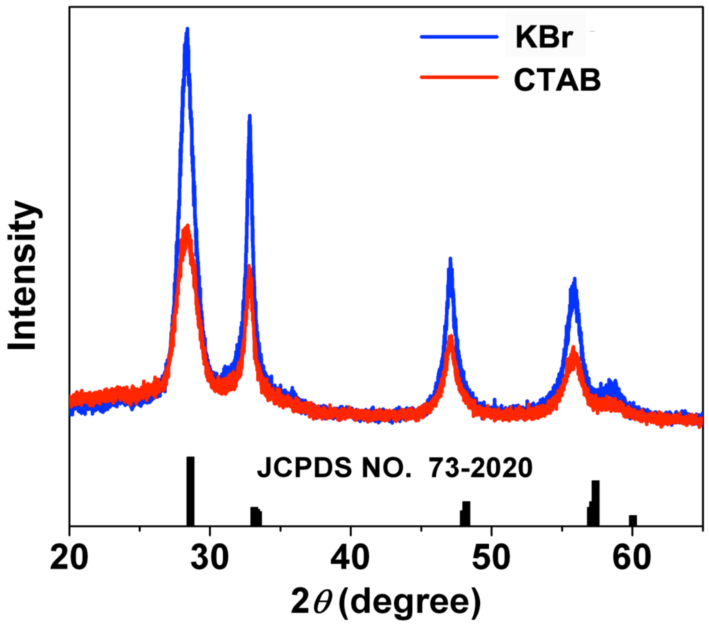

Supplementary Figure 1. XRD patterns of Bi_2WO_6 nanosheets prepared with KBr or CTAB assistance.

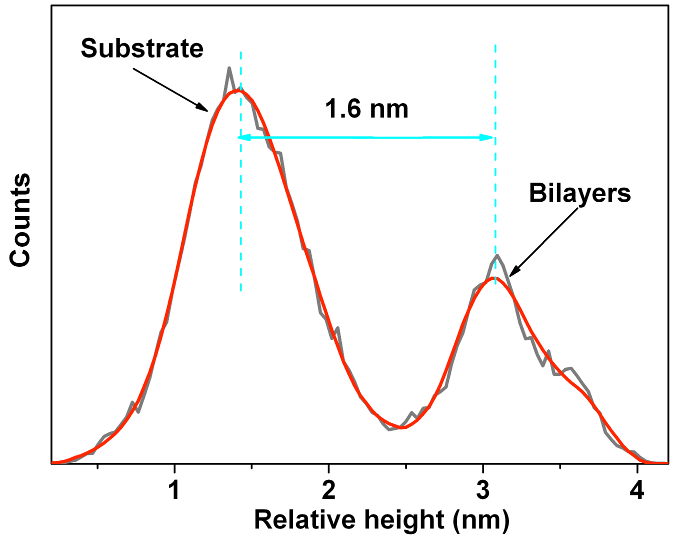

Supplementary Figure 2. Height histograms obtained from the AFM images of the Bi_2WO_6 sample prepared with KBr assistant (Figure 1f). It should be noted that the small monolayers are also observed in the AFM images. But it is difficult to distinguish the monolayers from substrate in the height histograms because of their small size and extra-thin thickness.

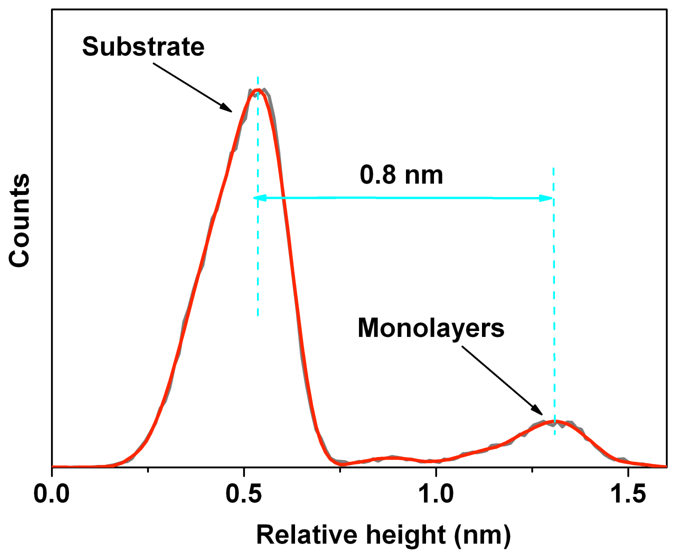

Supplementary Figure 3. Height histograms obtained from the AFM images of the monolayer Bi_2WO_6 prepared with CTAB assistant (Figure 1i).

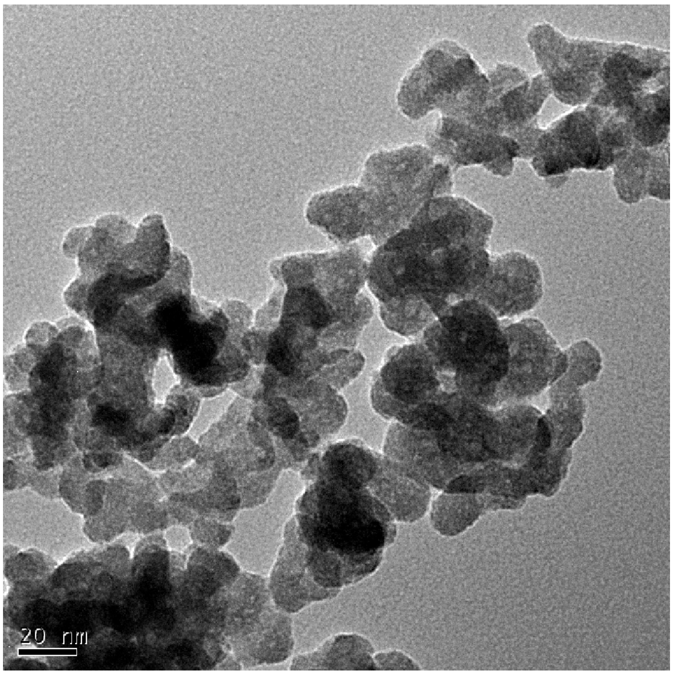

Supplementary Figure 4. TEM image of Bi_2WO_6 nanocrystals prepared without Br^- ions assistance.

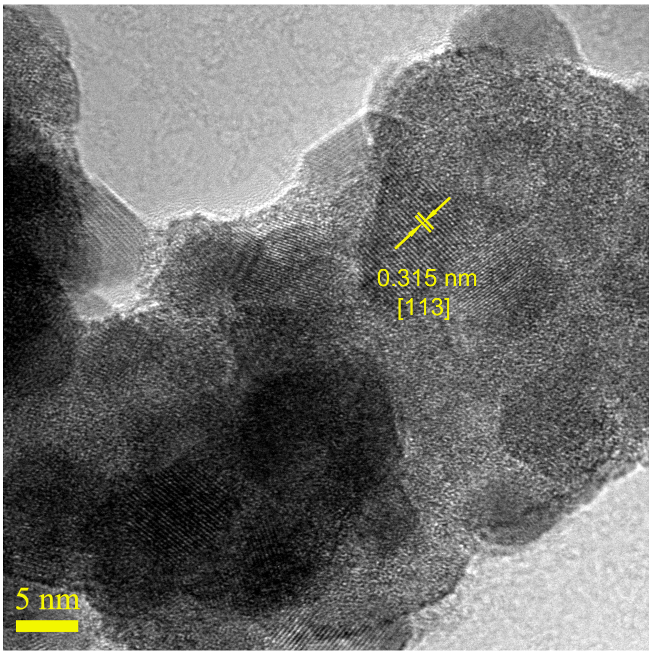

Supplementary Figure 5. HRTEM image of Bi_2WO_6 nanocrystals prepared without Br^- ions assistance.

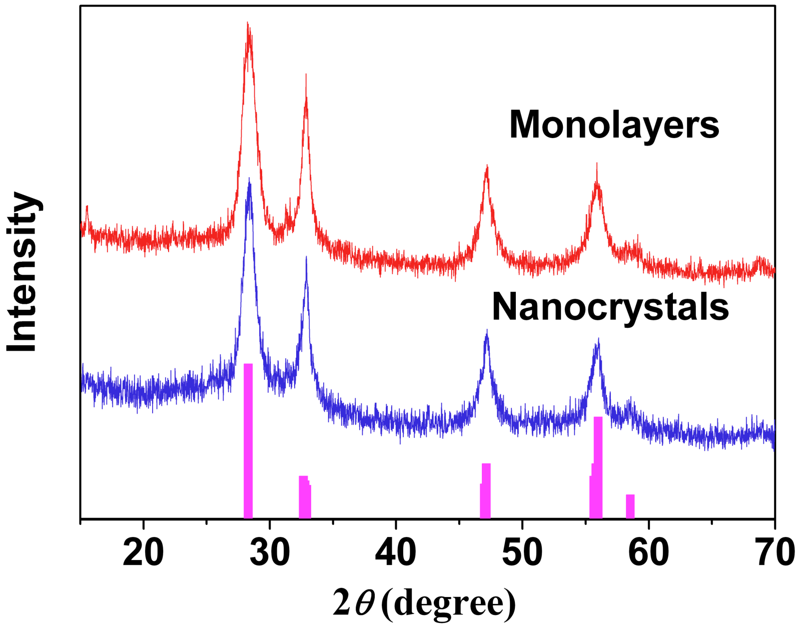

Supplementary Figure 6. XRD patterns of Bi_2WO_6 monolayers and nanocrystals

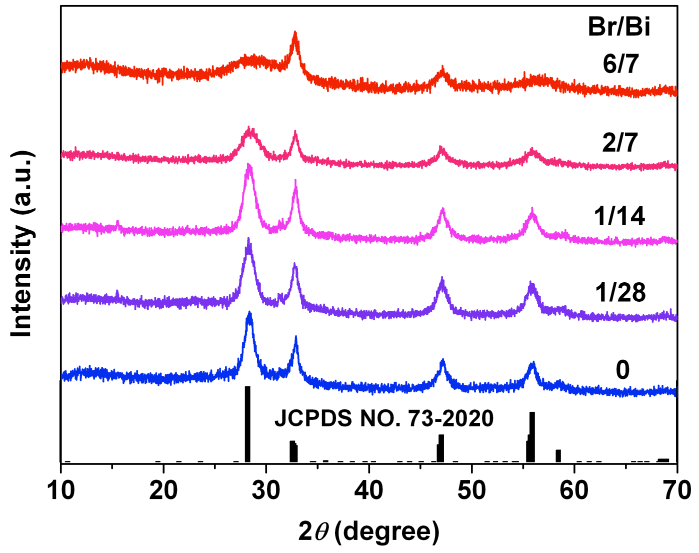

Supplementary Figure 7. The XRD patterns of Bi_2WO_6 samples prepared with various amounts of CTAB assistances (various Br/Bi atomic ratios). In the case of small amount of CTAB (Br/Bi atomic ratio ≤ 1/14), the Bi_2WO_6 samples show no obvious changes in the XRD patterns. But in the presence of high amounts of CTAB, the Bi_2WO_6 samples become poor in its crystallinity. Possibly that the large amounts of CTA^+ ions crowding on surface disfavors the growth of monolayer Bi_2WO_6 nanosheets.

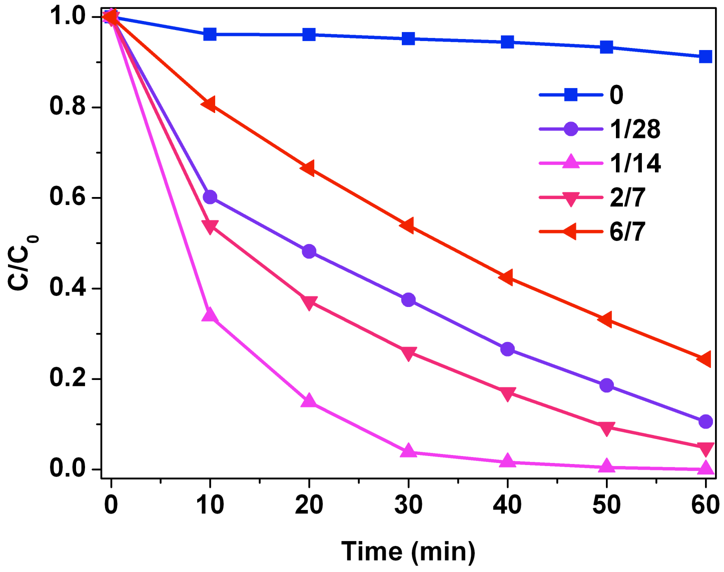

Supplementary Figure 8. The photodegradation performances of Bi_2WO_6 samples prepared with various amount CTAB added (Br/Bi atomic ratio in raw materials). Experiment condition: 50 mg catalyst, 80 mL RhB solution (10 μmol L^{-1}), a 500 W

halogen lamp with cut-off filters (420 nm ≤ λ ≤ 800 nm), no adsorption-desorption equilibrium prior irradiation. The Bi_2WO_6 nanoparticles obtained without Br^- ions assistance (Br/Bi = 0) show a low photodegradation activity. With small amount of CTAB (Br/Bi =1/14), the monolayer nanosheets was obtained and presented excellent activity. But large amount of CTAB induces the Bi_2WO_6 samples toward amorphous phase and thus gives low activity.

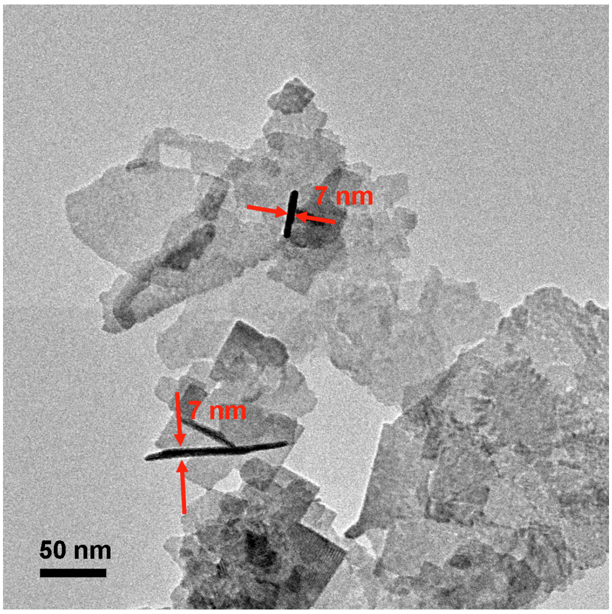

Supplementary Figure 9. TEM image of Bi_2WO_6 nanosheets prepared with large amount of KBr assistance (the Br/Bi atomic ratio is 10). Comparing with the Bi_2WO_6 sample prepared with Br/Bi = 20 shown in Fig. 2e-f, the more KBr in raw materials induced the thicker Bi_2WO_6 nanosheets.

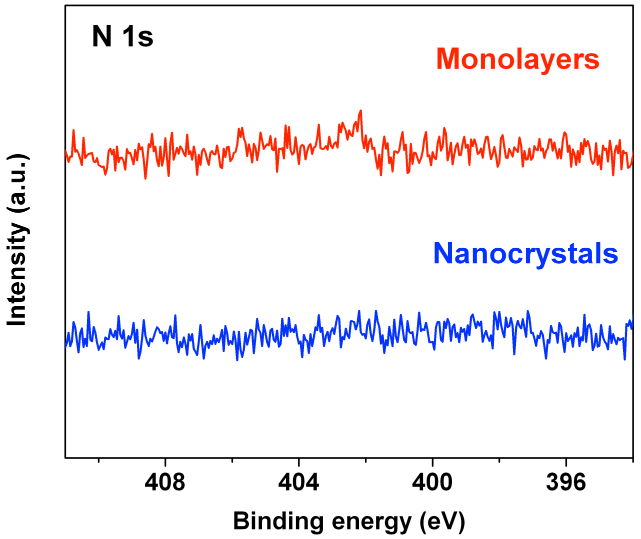

Supplementary Figure 10. N 1*s* XPS spectrum of the Bi_2WO_6 monolayers and nanocrystals. It is observed that the residual CTA^+ ions on the monolayers surfaces are rare, which implies that most of CTA^+ ions could be removed by washing after the preparation process.

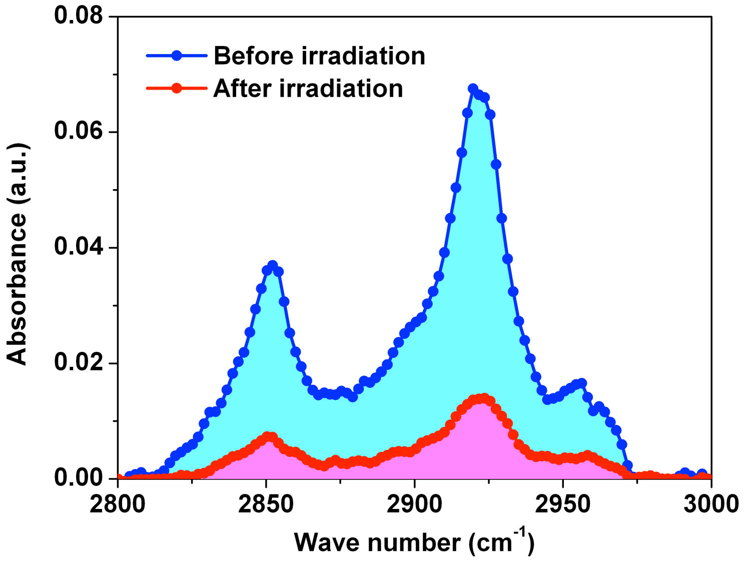

Supplementary Figure 11. FTIR spectra of the residual CTA^+ photodegradation under visible light. Experimental condition: 50 mg the Bi_2WO_6 monolayers dispersed in 80 mL water and then under visible light irradiation for one hour. After that the monolayers were collected and dried for IR test. Two bands at 2,852 and 2,922 cm^{-1}, characteristics of the C–H stretching mode, were used to detect the CTA^+ on the monolayers surfaces.[1] About 4/5 of the residual CTA^+ on the monolayers surfaces can be photodegraded in one hour.

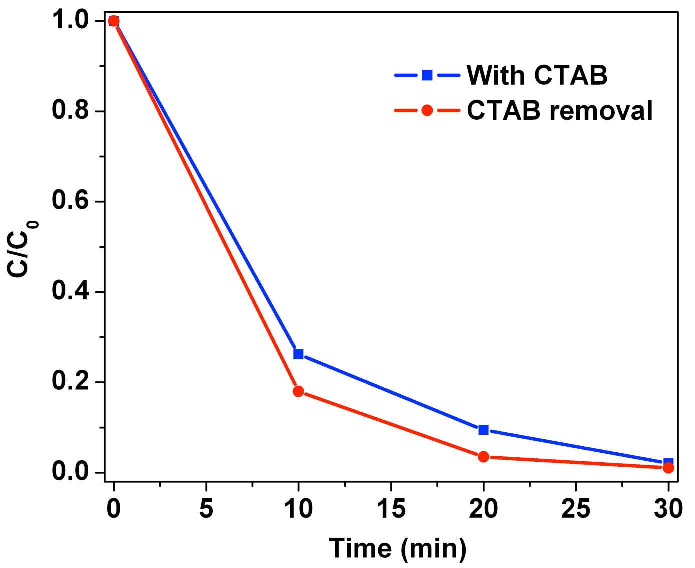

Supplementary Figure 12. Visible-light-driven photodegradation of RhB over the Bi_2WO_6 monolayers before and after removing the residual CTA^+ ions. Experimental condition: 50 mg catalyst, 80 mL RhB solution (10 μmol L^{-1}), 500 W halogen lamp with cut-off filters (420 nm ≤ λ ≤ 800 nm), no adsorption-desorption equilibrium process before irradiation. It is observed that the residual CTA^+ ions could slightly decrease the photodegradation activity of the monolayers due to the competitive photodegradation between the CTA^+ ions and the organic pollutants. However, the residual CTA^+ is rare and can be effectively removed during the photodegradation process, so the influence of CTA^+ on the photocatalytic activity of monolayers is almost negligible.

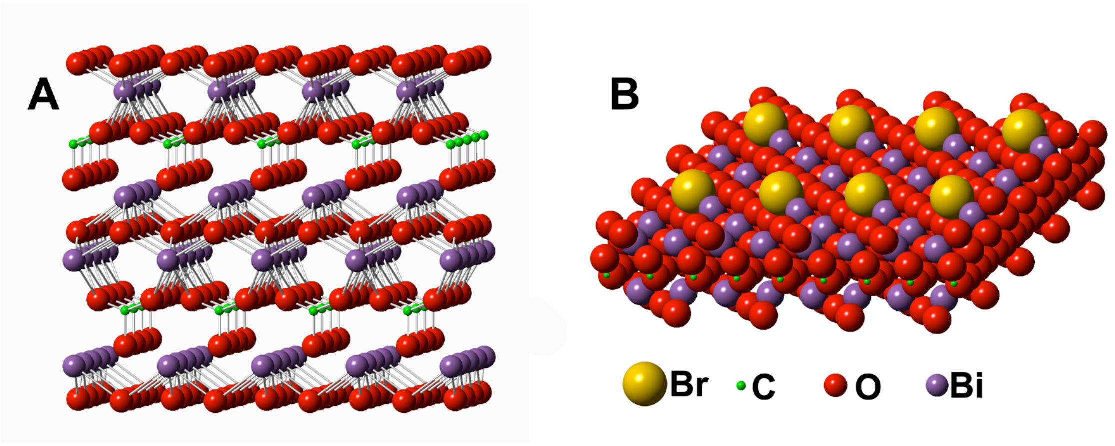

Supplementary Figure 13. (A) Crystal structure of $Bi_2O_2CO_3$. (B) Schematic illustration of the $Bi_2O_2CO_3$ monolayers prepared by the CTAB assisted self-assembly approach.

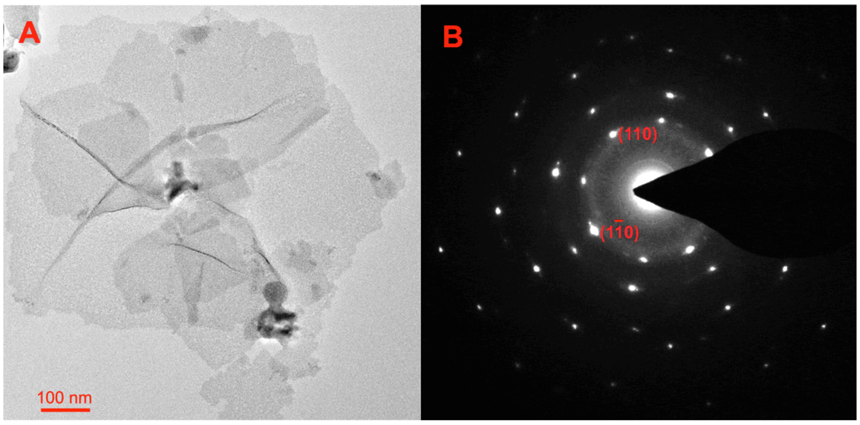

Supplementary Figure 14. (A)TEM image of $Bi_2O_2CO_3$ monolayers. (B) Corresponding selected area electron diffraction (SAED) pattern.

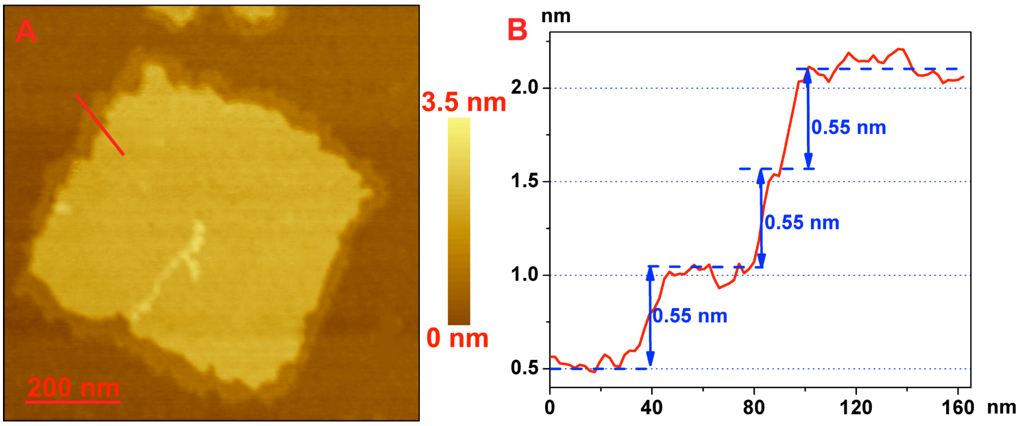

Supplementary Figure 15. (A) AFM image of $Bi_2O_2CO_3$ monolayers. (B) Corresponding height of the red line maked in (A).

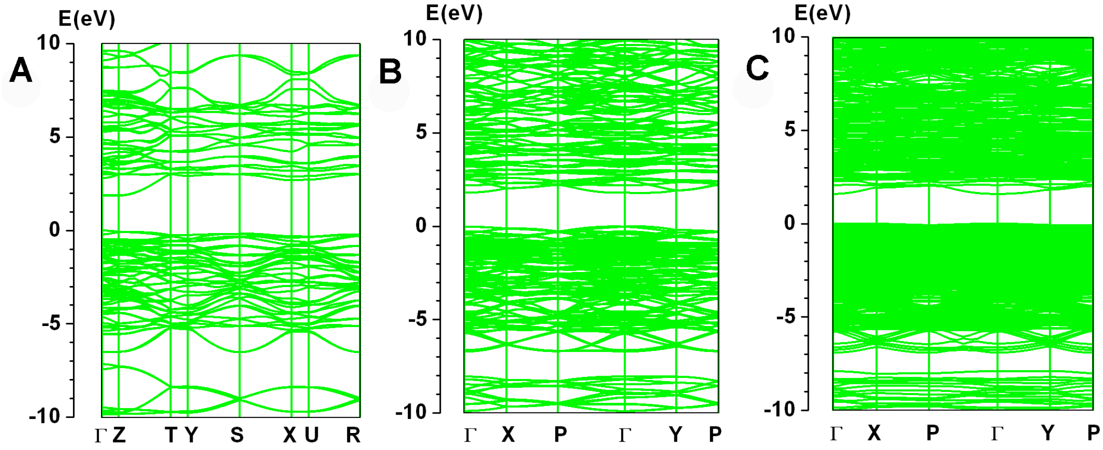

Supplementary Figure 16. Calculated band structures of Bi_2WO_6 bulk (A), pristine monolayers (B) and Br-decorated monolayers (C). The Fermi level is taken as the energy zero.

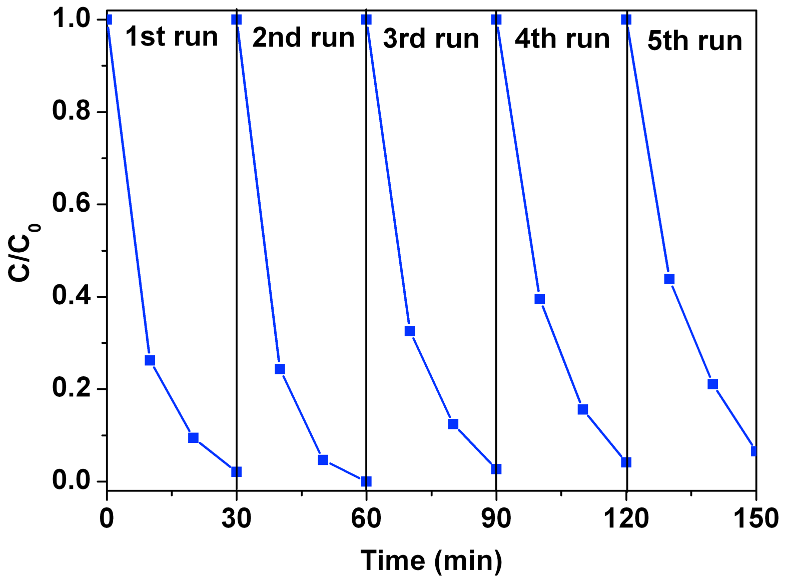

Supplementary Figure 17. Cycling runs of the photocatalytic degradation of RhB over the monolayer Bi_2WO_6 under visible light. Experiment condition: 50 mg catalyst, 80 mL RhB solution (10 μmol L^{-1}), 500 W halogen lamp with cut-off filters (420 nm ≤ λ ≤ 800 nm), no adsorption-desorption equilibrium process before irradiation. It is revealed that the monolayers can be efficiently recycled and reused without appreciable loss of activity.

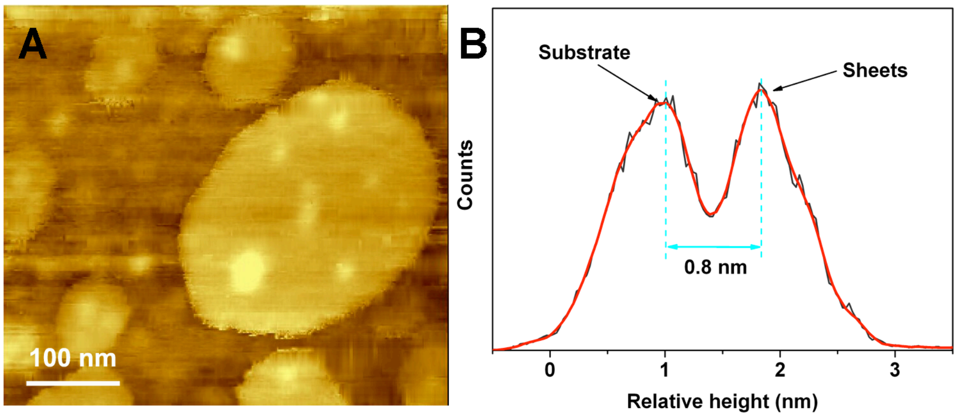

Supplementary Figure 18. (A) AFM image of the monolayer Bi_2WO_6 after the five cycle runs of photodegradation of RhB under visible light. (B) Height histograms obtained from the AFM image (A). The thickness of the monolayers presents no change after the five photocatalytic cycling tests, which attests that the structure of the monolayers is very stable.

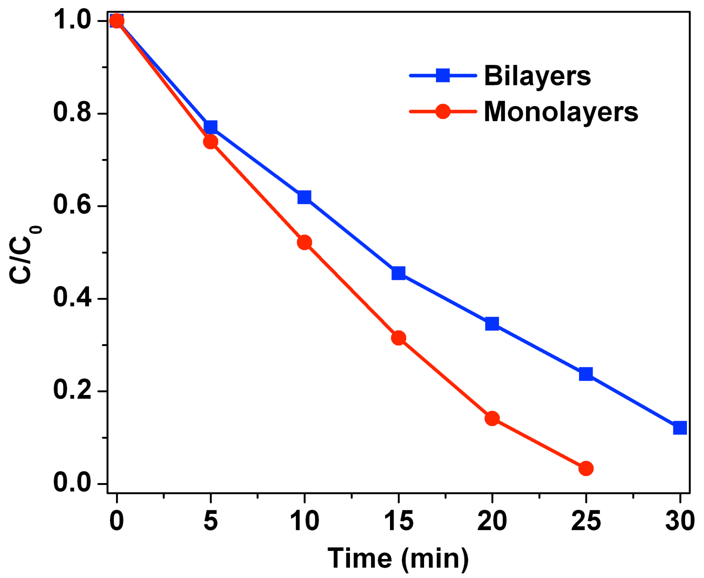

Supplementary Figure 19. Photodegradation of RhB over Bi_2WO_6 samples ($\lambda \geq 420$ nm). The sample prepared with KBr assistance containing some bilayers was marked the bilayers. Prior to the photocatalysis, the catalyst-dye solution was kept in the dark for 30 min to completely saturate the dye adsorption on the catalyst surface. The monolayers, as the thinnest Bi_2WO_6 materials, exhibit a much better photocatalytic activity than the bilayers.

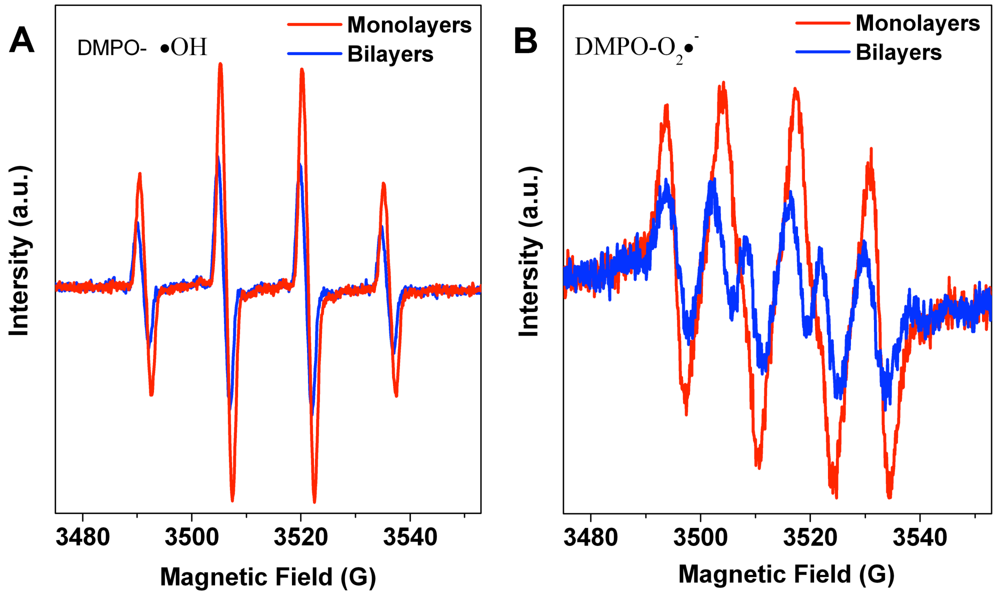

Supplementary Figure 20. ESR signals of the DMPO-•OH^- adducts and the DMPO-O_2•$^-$ adducts produced by the monolayers and the bilayers under visible-light irradiation ($\lambda \geq 420$ nm). The sample prepared with KBr assistance containing some

bilayers was marked the bilayers. The monolayers created more active oxygen species •OH and •O_2^- than the bilayers under visible-light irradiation, which confirms the monolayers with better photocatalytic performance than the bilayers.

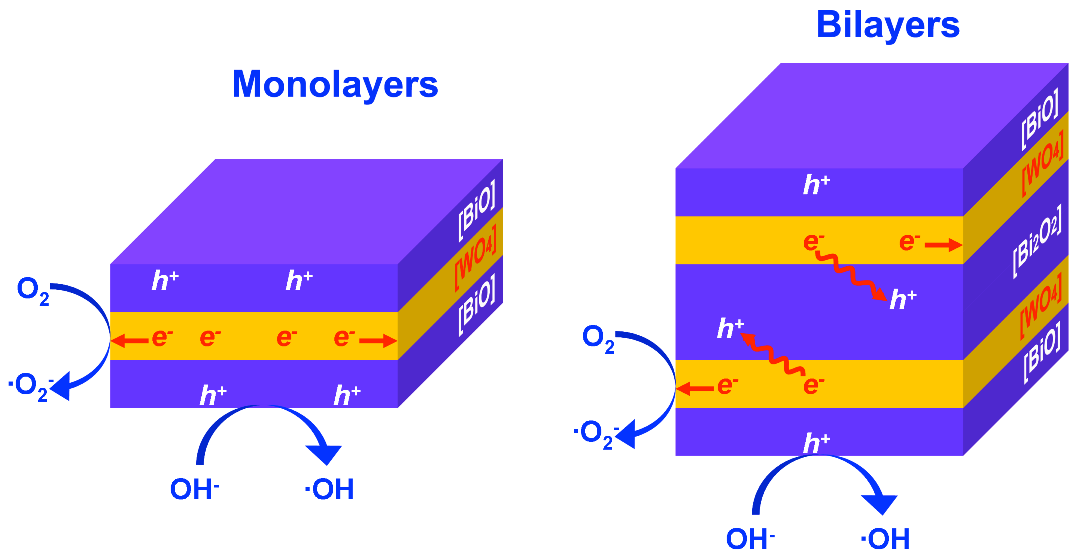

Supplementary Figure 21. Schematic illustration of the photoinduced charge transfer and separation on the monolayers and the bilayers after photoexcitation. For the bilayers, the holes are generated both on the surface layers and in the bulk. While the surface holes can be efficiently scavenged, the holes in the bulk can easily recombine with the photogenerated electrons. As a result, the monolayers possess more efficient charge separation than the bilayers, let alone multilayers.

Supplementary Table 1. Properties of the Bi_2WO_6 monolayers and nanocrystals

Sample	Band gap (eV)	Zeta potential at pH = 6 (mV)
Nanocrystals	2.9	27
Monolayers	2.7	-19

Supplementary References

1. Luisetto, I.; Pepe, F.; Bemporad, E., Preparation and characterization of nano cobalt oxide. *J. Nanopart. Res.* **10**, 59-67 (2008).
